# Interim guidelines for the assessment and treatment of pain in children with multiple sclerosis

**DOI:** 10.3389/fnins.2023.1235945

**Published:** 2023-09-14

**Authors:** Catherine Stratton, Areti Vassilopoulos, J. Nicholas Brenton, Kirsten Potter, Wendy Vargas, Heather Rumm, Andrea Bartels, Mary Bailey, Charles Odonkor, Sharon Stoll, E. William T. Zempsky, E. Ann Yeh, Naila Makhani

**Affiliations:** ^1^Department of Epidemiology, Dalla Lana School of Public Health, University of Toronto, Toronto, ON, Canada; ^2^Department of Pediatrics, Yale School of Medicine, New Haven, CT, United States; ^3^Child Study Center, Yale School of Medicine, New Haven, CT, United States; ^4^Division of Pediatric Neurology, Department of Neurology, University of Virginia Medical Center, Charlottesville, VA, United States; ^5^Department of Physical Therapy, Tufts University, Medford, MA, United States; ^6^Department of Neurology, Columbia University Vagelos College of Physicians and Surgeons, New York, NY, United States; ^7^Department of Neurology, New York-Presbyterian NYP/Columbia University Irving Medical Center, New York, NY, United States; ^8^Connecticut Chapter, National Multiple Sclerosis Society, Hartford, CT, United States; ^9^Department of Neurology, Yale School of Medicine, New Haven, CT, United States; ^10^Trinity Health of New England, Hartford, CT, United States; ^11^Department of Orthopedics and Rehabilitation, Yale School of Medicine, New Haven, CT, United States; ^12^Yale New Haven Health Old Saybrook Medical Center, Old Saybrook Medical Center, New Haven, CT, United States; ^13^Yale MS Center, North Haven, CT, United States; ^14^Division of Pain & Palliative Medicine, Connecticut Children’s Medical Center, Hartford, CT, United States; ^15^Department of Pediatrics, University of Connecticut School of Medicine, Farmington, CT, United States; ^16^Department of Paediatrics, Temerty Faculty of Medicine, University of Toronto, Toronto, ON, Canada; ^17^Division of Neuroscience and Mental Health, Department of Paediatrics (Neurology), Hospital for Sick Children, SickKids Research Institute, Toronto, ON, Canada

**Keywords:** pain, pediatrics, multiple sclerosis, clinical guideline, Delphi panel

## Abstract

**Introduction:**

Pain in multiple sclerosis (MS) is common, but literature on pain in children with MS remains scarce. Pain has physical, psychological, and social implications in MS, and both comprehensive assessment and interdisciplinary management approaches are needed. We sought to develop an interdisciplinary interim guideline for the assessment and management of pain in children with MS.

**Methods and materials:**

We convened a modified Delphi panel composed of 13 experts in pediatric and adult MS neurology, physiotherapy, pain, patient lived-experience, advanced practice nursing, psychology, physiatry, and MS research. A survey was sent to panelists for anonymous completion. The panel discussed survey themes extracted by the panel chair. The process was repeated twice.

**Results:**

Thirteen assessment and treatment recommendations were produced regarding pain in children with MS.

**Discussion:**

Future studies will assess implementation of these pain assessment and treatment guidelines in the clinical setting.

## 1. Introduction

Multiple sclerosis (MS) is an autoimmune disease associated with demyelination, neurodegeneration, and chronic inflammation of the central nervous system (Vargas-Lowy and Chitnis, 2012; Filippi et al., 2016; Brenton et al., 2020). While most often diagnosed in adulthood, approximately 2–10% of individuals with MS experience their first clinical symptom(s) before age 18 (Vargas-Lowy and Chitnis, 2012; Jancic et al., 2016; Narula, 2016; Nikolić et al., 2020).

The reported prevalence of pain in people with MS ranges from 29 and 86% (Jancic et al., 2016; Urits et al., 2019; Yilmazer et al., 2020). Several types of pain have been observed in people with MS. Among these, neuropathic pain is the most commonly reported, occurring in as many as 86% of patients (Urits et al., 2019; Yilmazer et al., 2020). Generalized back pain, tonic spasms or spasticity, L’Hermitte’s symptom, visceral pain, and trigeminal neuralgia are other types of pain that are also observed in adults with MS (Ferraro et al., 2017; Aboud and Schuster, 2019; Yilmazer et al., 2020). Similar pain presentations are also observed in children (Wang and Greenberg, 2018). The wide range of pain types and their varying presentations (Wang et al., 2017; Bosma et al., 2018; Urits et al., 2019) may make the assessment and management of pain challenging. Pain, however, affects the day to day lives of patients with MS. For example, pain flares reported in adults (Aboud and Schuster, 2019) are strongly correlated with quality of life (QoL) to a greater extent in people with MS than in people with other neurological conditions (Ferraro et al., 2017; Marck et al., 2017; Yilmazer et al., 2020).

Given the burden of pain in MS, comprehensive recommendations for children with MS are needed (Wang and Greenberg, 2018). Herein, we propose an interim guideline for pain assessment in children with MS based on the results of a modified Delphi process.

## 2. Materials and methods

### 2.1. Study design

We conducted a modified Delphi study to develop an interim guideline for the assessment and management of pain in children with MS. Our approach is modified because it included face-to-face meetings which were not included in the original Delphi methods (Dalkey and Helmer-Hirschberg, 1962). Measuring consensus expert opinion through Delphi and modified Delphi processes are models for establishing guidelines that has been applied for other conditions affecting children such as new onset refractory status epilepticus (NORSE), febrile infection related epilepsy syndrome (FIRES), and anti-NMDA receptor encephalitis (Hirsch et al., 2018; Nosadini et al., 2021; Wickström et al., 2022). It is especially useful for conditions for which there is limited research, such as pain assessment and management among children with MS (Wickström et al., 2022). We assessed expert opinions regarding pain metrics (e.g., pain frequency, pain intensity, pain sensation), pain assessment tools, intervention considerations, and application of interdisciplinary approaches to care. Consistent with other Delphi studies, consensus agreement was defined as at least 75% agreement (Diamond et al., 2014).

### 2.2. Ethics statement

The project was deemed exempt from review (#2000027448) by the Yale University Institutional Review Board (IRB).

### 2.3. Expert panel

The principal investigator (NM) and Delphi panel chair (CS) identified a sample of experts in the field of MS and/or pain research. Investigators recruited interdisciplinary representatives with the following expertise: child MS neurology, adult MS neurology, physiotherapy, pediatric pain medicine, patient lived-experience, nursing, social work and pediatric psychology, physiatry and rehabilitation medicine, and MS research. Thirteen experts participated in each round of this Delphi study. Informed consent was provided through the experts’ responses confirming participation via email. A social worker was replaced by a pediatric psychologist after Round 1 due to scheduling conflicts. Many panelists provided expertise in two areas (e.g., pediatric neurology and research). Six panelists work exclusively in a pediatric context.

### 2.4. Procedure

**Figure 1:**
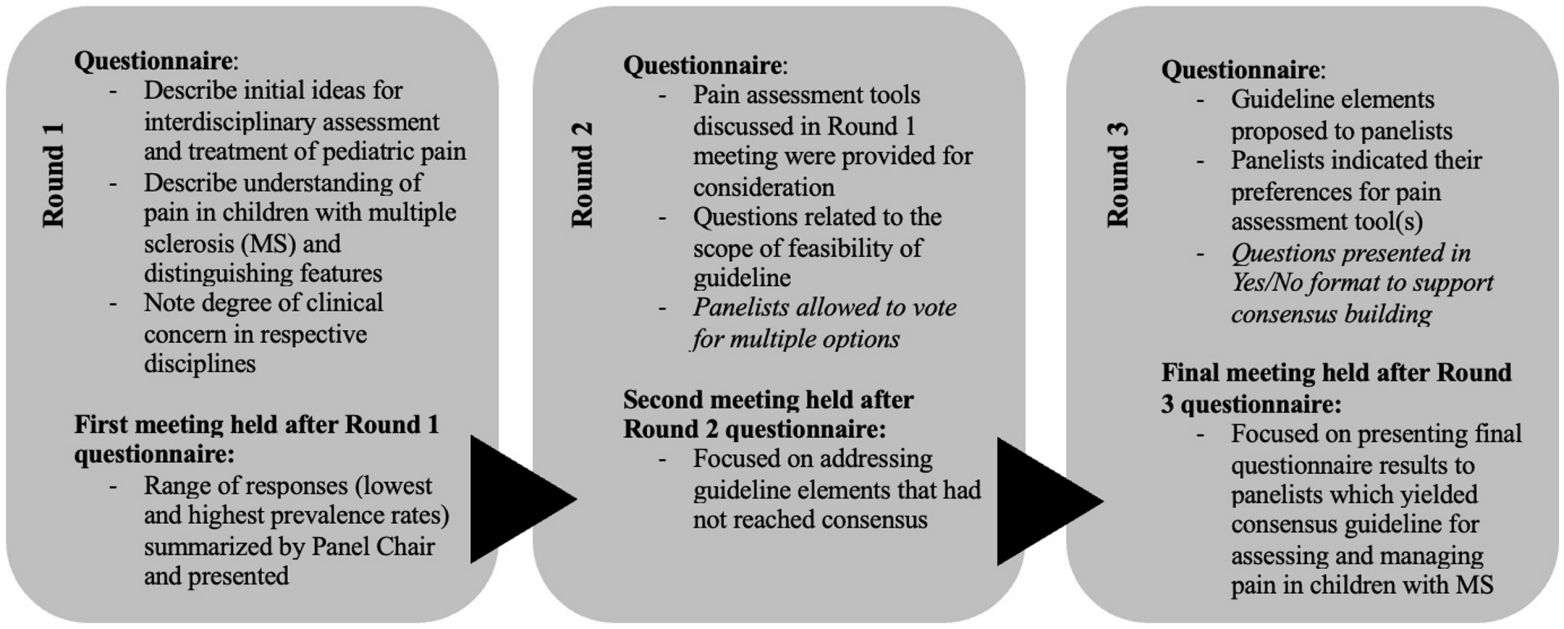
Summary of questionnaires for each round of the modified Delphi panel.

There were three rounds of Delphi surveys and panel discussions. For each round, a series of statements were sent to the panelists via electronic survey using REDCap which were followed by discussion rounds with the panel. The responses were anonymized except for questions asking panelists to describe their expert roles. All participants responded and were able to meet to discuss guidelines at each round. The number of respondents (N), the level of agreement (LA) and level of disagreement (LD) for the final recommendations are reported. A summary of the matters discussed in each round can be found in Figure 1.

## 3. Results

The Delphi panelists concluded that assessment of pain in children with MS involves three major considerations: (1) Children with MS report a variety of pain types, including neuropathic pain, spasticity-related pain, and various forms of headaches, (2) Pain symptoms in children with MS can vary widely with periods of remission followed by flares that impact quality of life and activities of daily life, and (3) Differences exist between children and adults in their ability to accept, manage, and articulate their pain’s characteristics and functional impact. Panelists estimated that recurrent, MS-related occurred in 25–50% of children in their own clinical practices. A summary of the assessment and treatment guidelines is in Table 1.

**Table 1:** Interim guideline recommendations.

Guideline criteria	Respondents in agreement *n* (%)	Respondents in disagreement *n* (%)
**Pain assessment**		
Pain will be assessed during all initial MS consultations.	13 (100.0)	0 (0.0)
Pain will be assessed upon follow-up as needed.	13 (100.0)	0 (0.0)
Patients should be asked to describe their pain’s sensation(s), intensity (e.g., mild, moderate, severe), and location(s) in their own words whenever possible.	13 (100.0)	0 (0.0)
A list of adjectives, such as through The Adolescent Pediatric Pain Tool (APPT), should be provided to describe pain’s sensation(s)/intensity if needed.	13 (100.0)	0 (0.0)
Terms such as “worst” and The Faces Pain Scale should be provided to describe their pain’s intensity if needed.	13 (100.0)	0 (0.0)
A body map diagram should be provided to describe pain’s location(s) if needed.	13 (100.0)	0 (0.0)
The impact of pain on activities of daily living (ADLs) can be assessed by the Functional Disability Index (FDI), Patient Reported Outcomes Measurement Information System (PROMIS) Pediatric Pain Interference Short Form and/or the Child Activity Limitations Interview (CALI-21).	13 (100.0)	0 (0.0)
Psychosocial concerns are brought forward quite frequently at MS appointments. Based on the initial consultation and follow-up assessment, referral for psychotherapy will be made if indicated.	13 (100.0)	0 (0.0)
Headache will be mentioned as an important symptom in some children with MS, but there will not be specific recommendations due to its complexity and unique treatment needs (meriting a separate guideline).	12 (92.3)	1 (7.7)
**Pain treatment**		
An interdisciplinary team of health care professionals who can provide holistic care for pain associated with MS is best practice.	13 (100.0)	0 (0.0)
Over-the-counter pharmaceuticals should be tried before introducing prescriptions medications.	13 (100.0)	0 (0.0)
When prescriptions are made, they should be agents targeted for a specific type of pain.	13 (100.0)	0 (0.0)
Psychosocial care should follow the assessment if indicated.	13 (100.0)	0 (0.0)

Factors not reaching consensus were not included in the final guideline.

### 3.1. Assessment guidelines

#### 3.1.1. Child-friendly assessment of pain locations, sensation, and intensity

##### 3.1.1.1. Recommendations

1. Patients should be asked to describe their pain intensity (e.g., mild, moderate, severe), and location(s) in their own words whenever possible (*N* = 13; LA = 100%; LD = 0%)
2. A list of adjectives can be provided such as through The Adolescent Pediatric Pain Tool (APPT) to describe pain sensation and intensity (*N* = 13; LA = 100%; LD = 0%)
3. A visual pain scale, such as the Faces Pain Scale, can be provided to describe their pain intensity (*N* = 13; LA = 100%; LD = 0%)
4. Body map diagrams can be provided to describe pain location(s) (*N* = 13; LA = 100%; LD = 0%)

The panelists agreed that asking patients to describe their pain in their own words was preferred whenever possible. Tools such as The Adolescent Pediatric Pain Tool (APPT) (ages 8–17 years old) (Jacob et al., 2014) may help patients and providers describe pain in a developmentally appropriate manner (e.g., instead of just listing “neuropathic pain,” list words such as, “burning”). Pain intensity descriptors such as, “typical” “worst” and “best” are helpful to frame current symptoms in the context of the patient’s typical experience. The Faces Pain Scale-Revised (FSP-R) (ages 4-17 years old), is a helpful option for younger children or children with learning disabilities (Hicks et al., 2001; Tsze et al., 2013). Panelists agreed that patients could show where their pain is located on a body map diagram (von Baeyer et al., 2011).

#### 3.1.2. Functionality and activities of daily living

##### 3.1.2.1. Recommendation

The impact of pain on activities of daily life (ADLs) can be assessed by the Functional Disability Index (FDI) (ages 8-18 years old) (Daffin et al., 2020); Patient Reported Outcomes Measurement Information System (PROMIS) Pediatric Pain Interference Short Form (ages 8–17 years old; parent proxy form available for children ages 5–17 years old) (Varni et al., 2010; PROMIS, 2023); and/or the Child Activity Limitations Interview (CALI-21) (ages 8–18 years old) (Palermo et al., 2008) (N = 13; LA = 100%; LD = 0%).

Considering the lack of a validated instrument for the pediatric MS setting and the variation of instrument preference amongst panelists, the panelists agreed all three instruments are viable options for assessing functionality and ADLs.

#### 3.1.3. Psychosocial

##### 3.1.3.1. Recommendation

Mental health screening should be completed, followed by appropriate referral for further psychological assessment, general psychotherapy, and/or health behavior intervention (*N* = 13; LA = 100%; LD = 0%).

Mental health is an important consideration in any comprehensive guideline for pain assessment and management, as pain is a biopsychosocial experience. There is a bidirectional relationship between chronic pain and mental health. A thorough assessment of school attendance, school-based supports, sleep quality and quantity, and functional disability is important. The panel recommended that psychological and psychosocial wellness should be assessed at the initial visit and follow-up visits, with referrals as appropriate.

#### 3.1.4. Headache

##### 3.1.4.1. Recommendation

Patients’ headache experience should be assessed, and a referral to a headache specialist should be made when needed (*N* = 13; LA = 92.3%; LD = 7.7%).

Children with MS presenting with headaches require a thorough headache evaluation to determine whether it is a primary headache disorder, secondary headache due to MS, or result of underlying non-MS pathology. Headache merits a separate guideline, given its complexities. The panelists agreed that clinicians should ask patients about headaches and can make a referral to a headache specialist if indicated.

#### 3.1.5. Frequency of pain assessment

##### 3.1.5.1. Recommendations

1. Pain will be assessed during all initial MS consultations (*N* = 13; LA = 100%; LD = 0%)
2. Pain will be assessed in follow-up visits as needed (*N* = 13; LA = 100%; LD = 0%)

Panelists agreed that pain should be assessed in all children with MS as part of the initial clinical assessment. Pain assessment questionnaires could be sent to patients ahead of time for electronic completion at home to promote efficiency of pain assessment in visit.

### 3.2. Treatment guidelines

#### 3.2.1. Holistic care team

##### 3.2.1.1. Recommendation

The treatment of pain in children with MS requires an interdisciplinary approach (*N* = 13; PA = 100%; PD = 0%).

Interdisciplinary care is essential for pain management. An interdisciplinary team may involve physicians, physiotherapists, nurses, psychologists, and social workers. Patients are important decision makers and members of their own pain treatment team.

#### 3.2.2. Pharmaceutical

##### 3.2.2.1. Recommendations

1. In general, over-the-counter pharmaceuticals should be tried before introducing prescription medications (*N* = 13; LA = 100%; LD = 0%)
2. Wherever possible, therapeutic agents should be targeted for the patient’s specific type of pain (*N* = 13; LA = 100%; LD = 0%)

Panelists agreed that, whenever possible, providers should first recommend over-the-counter analgesics, such as acetaminophen or ibuprofen. Prescription agents that target specific pain symptoms including anticonvulsants (e.g., gabapentin), serotonin and norepinephrine reuptake inhibitor (e.g., duloxetine), or tricyclic antidepressants (e.g., amitriptyline) should be employed if over-the-counter agents are ineffective or not indicated for the pain type (e.g., neuropathic pain).

#### 3.2.3. Psychosocial

##### 3.2.3.1. Recommendation

Behavior pain treatments should be recommended and for all patients with pain at baseline and continued as indicated (*N* = 13; LA = 100%; LD = 0%).

Adopting a biopsychosocial approach to pain management has been demonstrated to lead to improved outcomes among patients with chronic pain (Eucker et al., 2022). All panelists agreed that incorporating non-pharmaceutical treatments into a balanced treatment plan should be encouraged. These can include cognitive behavioral therapy for pain, biofeedback-assisted relaxation training, guided imagery, and mindfulness.

## 4. Discussion

Herein, we propose interim guidelines for the assessment and treatment of pain in children with MS which we developed using a modified Delphi panel. There are different types of pain in MS, which may fluctuate due to the progressive and relapsing nature of the disease (Jeong et al., 2019). Repeated, thorough assessments may allow for early detection and appropriate pain management intervention for children with MS which has been observed in adults with MS being assessed for neuropathic pain (Ferraro et al., 2017).

Pain treatment should be specific to the pain presentation and may include pharmaceutical and non-pharmaceutical. In a study in adults, approximately 15% of people with MS were on a pharmaceutical regimen for chronic pain (Ferraro et al., 2017). Gabapentin (33.3%), pregabalin (28.0%), duloxetine (21.0%) and amitriptyline (16.0%) were the most prescribed medications (Ferraro et al., 2017). Another 5.6% of patients had been prescribed cannabinoid-based medication for spasticity, 42.1% were taking non-steroidal anti-inflammatory drugs as treatment, and 3.1% patients were prescribed and utilized opioid medication (Ferraro et al., 2017). Slightly more than 10% of patients were prescribed greater than one medication for their pain symptoms (Ferraro et al., 2017). The optimal treatment of different pain types in children warrants further study.

Pain can interfere with physical, social, and psychological domains of functioning, potentially to a greater extent than other neurological disorders, which highlights the need for an interdisciplinary approach (Drulovic et al., 2015; Huang et al., 2017; Yilmazer et al., 2020). One study found that pain interference is associated with changes in the pain-processing connectome as detected by reduced beta power on magnetoencephalography (MCG), particularly in the thalamus and posterior insula, suggesting a potential biological mechanism for the pain experience in MS (Kim et al., 2018). People with MS experience greater pain-related interference in daily life the longer they have the disease (Yilmazer et al., 2020), highlighting the importance of recognizing and treating pain early in pediatric MS to support good quality of life. Several psychosocial treatment modalities have been studied in adult MS patients and other chronic pain populations which merit further study in the pediatric MS population. These include cognitive behavioral therapy (Ostojic et al., 2021), biofeedback-assisted relaxation training (Ostojic et al., 2021), and mindfulness (Senders et al., 2018).

Our study has some limitations. First, all panelists were from academic institutions in North America. This could affect generalizability of the guideline across cultures. Of note, the recommended instruments are available in an array of languages to allow for broad use. Further, the recommended instruments have not been validated in this population. However, the instruments have been validated in several pediatric populations with similar risk factors for pain as children with MS, including abdominal pain, musculoskeletal pain syndromes, back pain, headache, chronic kidney disease-related pain, Crohn’s disease-related pain, amongst others (Palermo et al., 2008; Kashikar-Zuck et al., 2011; Forrest et al., 2020). Lastly, the panel did not include a pediatric MS patient, though we did include an adult patient.

## 5. Conclusion

Pain in children with MS may negatively impact quality of life. Therefore, these proposed guidelines are essential for identifying, assessing, and treating pain in this population. Chronic pain is multifaceted, making interdisciplinary care critical. Future studies could assess the acceptability of the proposed guidelines amongst children with MS, the feasibility of implementing the guidelines in the clinical setting, as well as the validation of the proposed assessment tools in childhood MS. Other mental health aspects of pain are important to evaluate, but beyond the scope of the present study. Future guidelines should provide recommendations for psychological and social assessment, as well as a comprehensive screening battery involving pain, psychological, and social assessments.

## Data availability statement

The raw data supporting the conclusions of this article will be made available by the authors, without undue reservation.

## Author contributions

CS and NM designed the study. CS, AV, and NM drafted the initial manuscript. All authors reviewed the manuscript for important intellectual content and contributed edits.
